# (Epi)genetic defects of *MKRN3* are rare in Asian patients with central precocious puberty

**DOI:** 10.1038/s41439-019-0039-9

**Published:** 2019-01-21

**Authors:** Erina Suzuki, Hirohito Shima, Masayo Kagami, Shun Soneda, Toshiaki Tanaka, Shuichi Yatsuga, Junko Nishioka, Yuji Oto, Toshiya Kamiya, Yasuhiro Naiki, Tsutomu Ogata, Yasuko Fujisawa, Akie Nakamura, Sayaka Kawashima, Shuntaro Morikawa, Reiko Horikawa, Shinichiro Sano, Maki Fukami

**Affiliations:** 1 Department of Molecular Endocrinology, National Research Institute for Child Health and Development, Tokyo, Japan; 2 Department of Pediatrics, St. Marianna University School of Medicine, Kawasaki, Japan; 3 Tanaka Growth Clinic, Tokyo, Japan; 4 Department of Pediatrics and Child Health, Kurume University School of Medicine, Kurume, Japan; 5 Department of Pediatrics, Saitama Medical Center, Dokkyo Medical University, Saitama, Japan; 6 Department of Pediatrics, JA Mie Kouseiren Matsusaka Central General Hospital, Matsusaka, Japan; 7 Division of Endocrinology and Metabolism, National Center for Child Health and Development, Tokyo, Japan; 8 Department of Pediatrics, Hamamatsu University School of Medicine, Hamamatsu, Japan; 9 Department of Pediatrics, Hokkaido University School of Medicine, Sapporo, Japan; 10 Hamamatsu Medical Center, Hamamatsu, Japan

**Keywords:** Endocrinology, Medical genetics

## Abstract

We sequenced *MKRN3*, the major causative gene of central precocious puberty in Western countries, in 24 Japanese or Chinese patients and examined the DNA methylation and copy-number statuses of this gene in 19 patients. We identified no (epi)genetic defects except for one previously reported mutation. These results, together with reports from Korea, indicate that *MKRN3* defects are rare in Asian populations. The ethnic differences likely reflect Western country-specific founder mutations and the rarity of de novo mutations.

Central precocious puberty (CPP) is a rare multifactorial disorder caused by an age-inappropriate secretion of the gonadotropin-releasing hormone from the hypothalamus^1^. CPP can occur as a result of monogenic mutations, although it is frequently associated with brain lesions, such as tumor and injury^1^. Thus far, a few genes, including *KISS1R*, *KISS1*, *PROKR2*, and *NR0B1*, have been reported as causative genes for CPP^1–3^. In addition, two imprinted genes, *MKRN3* and *DLK1*, have recently been implicated in the development of CPP^4,5^. Mutations in *MKRN3* and *DLK1* cause CPP when they reside on paternally derived alleles. The association between epigenetic defects of *MKRN3* or *DLK1* and CPP has yet to be determined.

Previous studies in Western countries have identified pathogenic *MKRN3* mutations in 9–46% of familial cases and 3–20% of sporadic cases with CPP (Table 1), indicating that these mutations play an important role in the etiology of CPP. In contrast, Lee et al. identified pathogenic *MKRN3* mutations only in one of 260 Korean patients with CPP^6^. Likewise, Jeong et al. reported the lack of pathogenic *MKRN3* mutations in 26 Korean patients with familial CPP^7^. These data indicate that there may be an ethnic difference in the frequency of *MKRN3* mutations in CPP patients. However, *MKRN3* mutation analyses have rarely been performed in Asian countries other than Korea, except for our previous study in which *MKRN3* mutations were identified in one of 15 Japanese patients^8^. Moreover, since Lee et al.^6^ and Jeong et al.^7^ did not examine DNA methylation defects or copy-number alterations of *MKRN3*, these abnormalities may be hidden in their patients.

**Table 1:** Previous reports of large-scale *MKRN3* mutation screening on patients with central precocious puberty

Frequency of pathogenic *MKRN3* mutations^a^	Identified *MKRN3* mutations	Location of the hospital	Reference
Familial cases			
5/15 (33%)	c.482dupC (p.Ala162Glyfs*15)^b^, c.1095G > T (p.Arg365Ser), and other mutations	Multiple Western countries	Abreu et al.^4^
2/6 (33%)	c.482dupC (p.Ala162Glyfs*15)^b^, c.331G > T (p.Glu111*)	Germany	Schreiner et al.^10^
5/17 (29%)	c.482dupC (p.Ala162Glyfs*15)^b^, c.982C > T (p.Arg328Cys), and other mutations	Brazil	Bessa et al.^11^
13/28 (46%)	c.482dupC (p.Ala162Glyfs*15)^c^, c.802_803delAT (p.Met268Valfs*23), c.982C > T (p.Arg328Cys), and other mutations	Multiple Western countries	Simon et al.^12^
2/23 (8.7%)	c.1229G > A (p.Cys410*), c.478_485delCCCCCGGC (p.Pro160Cysfs*14)^d^	Italy	Grandone et al.^13^
2/2 (100%)	c.441delG (p.His148Thrfs*23)^e^, c.802_803delAT (p.Met268Valfs*23)	Turkey	Simsek et al.^14^
1/10 (10%)	c.632_650delCCTACCGGGGCCGCTGGGTinsTGGGC (p.Pro211Leufs*16)^f^	Turkey	Aycan et al.^15^
0/26 (0%)	None	Korea	Jeong et al.^7^
Sporadic cases			
1/18 (6%)	c.737A > G (p.Tyr246Cys)	Multiple Western countries	Simon et al.^12^
1/20 (5%)	c.203G > A (p.Arg68His)	Spain	Ortiz-Cabrera et al.^16^
1/37 (3%)	c.982C > T (p.Arg328Cys)	Italy	Grandone et al.^13^
8/215 (4%)	c.482delC (p.Pro161Argfs*10), c.482dupC (p.Ala162Glyfs*15)^g^, and other mutations	Brazil	Macedo et al.^17^
2/10 (20%)	c.1053_1056delACAG (p.Arg351Serfs*44), c.482delC (p.Pro161Argfs*10)	Brazil	Dimitrova-Mladenova et al.^18^
1/29 (3%)	c.1034G > A (p.Arg345His)	Denmark	Känsäkoski et al.^19^
1/260 (0.3%)	c.841C > T (p.Gln281*)	Korea	Lee et al.^6^

^a^The denominators indicate the number of families/patients examined, and the numerators represent the number of families/patients positive for pathogenic *MKRN3* mutations

^b^This substitution was initially described as c.475_476insC (p.Ala162Glyfs*14)

^c^This substitution was initially described as c.482insC (p.Ala162Glyfs*15)

^d^This substitution was initially described as c.477_485del (p.Pro160Cysfs*14)

^e^This substitution was initially described as c.441_441delG (p.His148Thrfs*23)

^f^This substitution was initially described as c.630_650delinsGCTGGGC (p.Pro211Leufs*16)

^g^This substitution was initially described as c.482_483insC (p.Pro161Argfs*16)

Here we searched for genetic and epigenetic defects of *MKRN3* in Japanese and Chinese patients with etiology-unknown CPP. Nucleotide substitutions were analyzed in 24 (22 Japanese and 2 Chinese) patients, whereas DNA methylation defects and copy-number alterations were examined only in 19 Japanese patients for whom we could obtain a sufficient amount of genomic DNA. This study was approved by the Institutional Review Board Committee of the National Research Institute for Child Health and Development and performed after obtaining written informed consent. All patients satisfied the following criteria: (i) early pubertal onset (in boys, testicular enlargement before 9 years of age, pubic hair before 10 years of age, or axillary hair/voice change before 11 years of age; in girls, breast budding before 7.5 years of age, pubic hair before 8 years of age, or menarche before 10.5 years of age); (ii) increased blood levels of gonadotropin and sex hormone; (iii) normal findings in brain magnetic resonance imaging; and (iv) no pathogenic mutations in *DLK1*, *KISS1R*, *KISS1*, *PROKR2*, or *NR0B1*. Patients with congenital malformation syndromes and those with chronic disorders that may affect hormone secretion were excluded from this study. Patients had no apparent family history of early puberty.

First, we searched for *MKRN3* sequence variations in 24 patients. Thirteen patients were previously subjected to whole-exome sequencing using a Nextera Rapid Capture Exome Kit (HiSeq SBS Kit v4-HS Illumina, San Diego, CA, USA) and a HiSeq 2500 sequencer (Illumina)^8^. The remaining 11 patients were first examined in the present study; targeted sequencing of their DNA was performed for 148 genes using the HaloPlex HS Target Enrichment System (Design ID 40350-1451214604; Agilent Technologies, Palo Alto, CA, USA) and a MiSeq sequencer (Illumina). Sequence data of the 24 patients were analyzed as described previously^3^. We focused on nonsynonymous variants in the coding region and intronic substitutions affecting splice sites of *MKRN3*. Variants whose frequency in the Japanese general population [the ExAC browser (http://exac.broadinstitute.org/) and the Human Genetic Variation Browser (http://www.hgvd.genome.med.kyoto-u.ac.jp/)] is more than 1% were excluded as polymorphisms.

Next, we conducted DNA methylation and copy-number analyses for 19 patients. The DNA methylation status of seven CpG sites at the *MKRN3* locus was examined by pyrosequencing using a previously described method^9^. Copy-number alterations of *MKRN3* were analyzed by real-time PCR using a TaqMan Copy Number Assay Kit (*MKRN3*, Hs02079798; internal control, 440332; ThermoFisher Scientific, Tokyo, Japan) according to the manufacturer’s instructions.

Consequently, rare nucleotide substitutions of *MKRN3* were not detected in the 24 patients except for one female patient with c.684dupA (p.Glu229Argfs*3), who has been reported previously^8^. Moreover, DNA methylation statuses were comparable between the patients and control individuals (Fig. 1). Similarly, copy-number analysis identified no deletions or duplications of *MKRN3* in all patients examined.

**Fig. 1:**
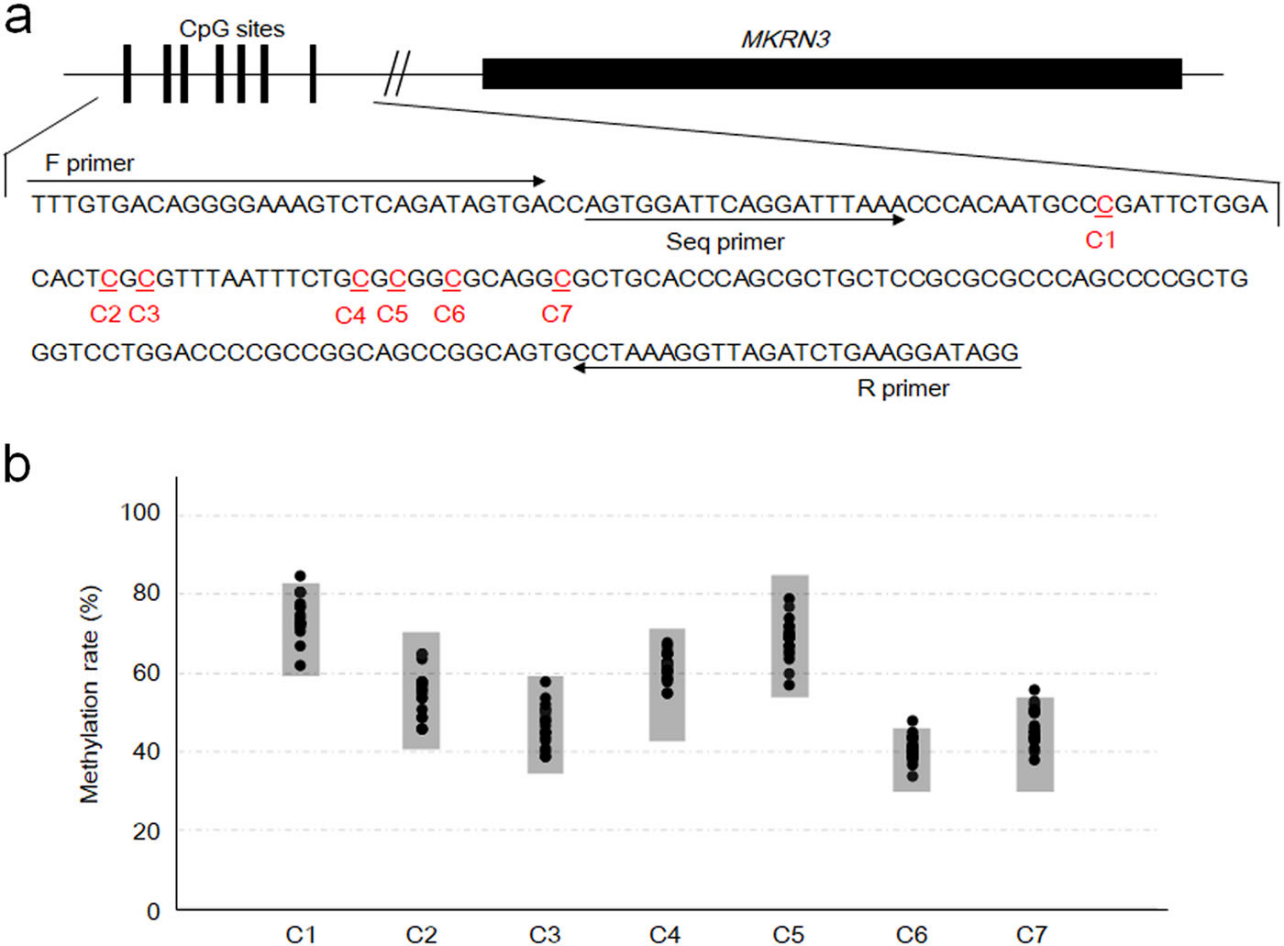
DNA methylation analysis of 19 patients. **a** Genomic structure of *MKRN3* and flanking CpG sites. C1–C7 represent cytosines at the CpG sites in the differentially methylated region. F and R primers were used for PCR amplification, and the Seq primer was used for pyrosequencing. This figure is not drawn to scale. **b** Results of methylation analysis. Black dots represent the methylation status of the patients. Gray shaded areas indicate reference ranges obtained from 49 control individuals

The results of this study expand the prior notion of Lee et al.^6^ and Jeong et al.^7^ to suggest that genetic and epigenetic defects in *MKRN3* are relatively rare in Asian CPP patients. Although underlying factors of the ethnic difference in the frequency of *MKRN3* mutations remain to be determined, the relatively high frequency in Western countries possibly reflects the presence of multiple founder mutations. Indeed, c.482delC (p.Pro161Argfs*10), c.482dupC (p.Ala162Glyfs*15), c.802_803delAT (p.Met268Valfs*23), c.982C > T (p.Arg328Cys), and c.1095G > T (p.Arg365Ser) have been repeatedly identified in patients from these countries (Table 1). In this regard, since there have been no reports of de novo *MKRN3* mutations in CPP patients, the de novo occurrence of *MKRN3* substitutions seems to be an exceptional event. Moreover, our data suggest that DNA methylation defects and copy-number alterations of *MKRN3* play only a minor role in the development of CPP, if at all.

In conclusion, the results of this study, together with two reports from Korea^6,7^, indicate that (epi)genetic defects of *MKRN3* represent only a minor cause of CPP in Asian populations. The presence of multiple founder mutations in Western countries as well as the rarity of de novo occurrences of intragenic nucleotide substitutions likely underlies the ethnic differences in the frequency of *MKRN3* mutations in CPP cases.

## Data Availability

The relevant data from this Data Report are hosted at the Human Genome Variation Database at 10.6084/m9.figshare.hgv.2525
